# Van der Woude Syndrome with Short Review of the Literature

**DOI:** 10.1155/2014/871460

**Published:** 2014-06-22

**Authors:** Pallavi K. Deshmukh, Kiran Deshmukh, Anand Mangalgi, Subhash Patil, Deepa Hugar, Saraswathi Fakirappa Kodangal

**Affiliations:** ^1^Department of Oral Medicine and Radiology, H.K.E.S.'s S. N. Institute of Dental Sciences and Research, Gulbarga, Karnataka 585103, India; ^2^Department of Otorhinolaryngology, M. R. Medical College, Gulbarga, Karnataka 585103, India; ^3^Department of Oral and Maxillofacial Surgery, H.K.E.S.'s S. N. Institute of Dental Sciences and Research, Gulbarga, Karnataka 585103, India; ^4^Department of Community and Preventive Dentistry, H.K.E.S.'s S. N. Institute of Dental Sciences and Research, Gulbarga, Karnataka 585103, India; ^5^Department of Oral Pathology and Microbiology, H.K.E.S.'s S. N. Institute of Dental Sciences and Research, Gulbarga, Karnataka 585103, India

## Abstract

Van der Woude syndrome (VWS) is a rare autosomal dominant condition with high penetrance and variable expression. Clinical manifestation of this autosomal dominant clefting syndrome includes bilateral midline lower lip pits, cleft lip, and cleft palate along with hypodontia. These congenital lip pits appear as a malformation in the vermilion border of the lip, with or without excretion. Discomfort caused by spontaneous or induced drainage of saliva/mucus when pressure is applied or during a meal as well as poor aesthetic match is one of the main complaints of patients with congenital lip fistula. The pits are treated by surgical resection. Dentists should be aware of the congenital lip pits as in Van der Woude syndrome because they have been reported to be associated with a variety of malformations or other congenital disorders. Here, the authors report a rare case of Van der Woude syndrome with short review of the literature.

## 1. Introduction

VWS also referred to in the literature as autosomal dominant inherited clefting syndrome is a rare congenital syndrome first described by Demarquay in 1845. VWS clinically presents with congenital lip pits. These lip pits occur on paramedian portion of the vermillion border of the lip. In VWS, congenital lip pits occur in concurrence with cleft lip and/or cleft palate and represent the most common clinical problem occurring in 80% of the patients [1]. Lip pits result due to notching of the lips at an early stage of development with fixation of tissues at the base of the notch or they may result from a failure of complete union of embryonic lateral sulci of lip [2]. The other associated features of Van der Woude syndrome which may or may not be present are hypodontia, hypoplasia, ankyloglossia, high arched palate, limb anomalies, congenital heart defects, and so forth [2, 3]. Most cases have been associated with deletion of chromosome 1q32–q41, but an extra chromosomal locus at 1p34 has been identified [2].

## 2. Case Report

**Figure 1:**
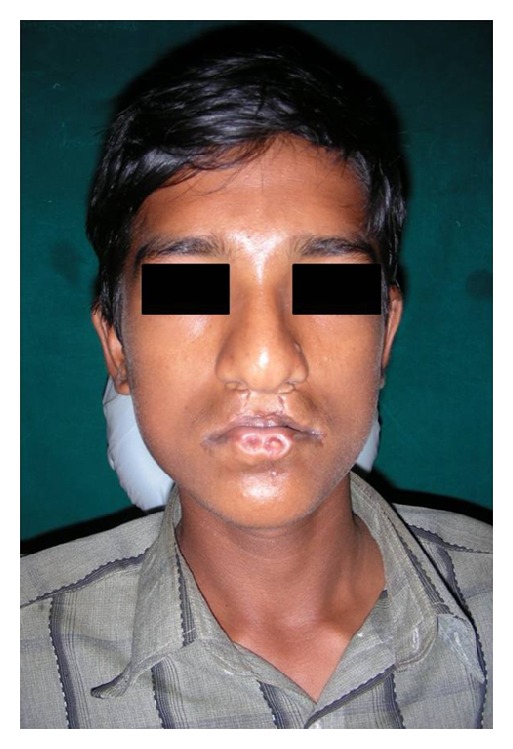
Frontal view of the patient showing midface retrusion and a repaired bilateral upper cleft lip.

**Figure 2:**
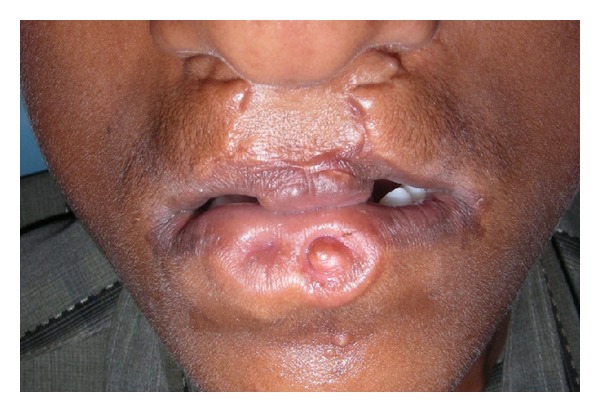
Close-up view showing two bilaterally symmetrical lip pits on the lower lip.

**Figure 3:**
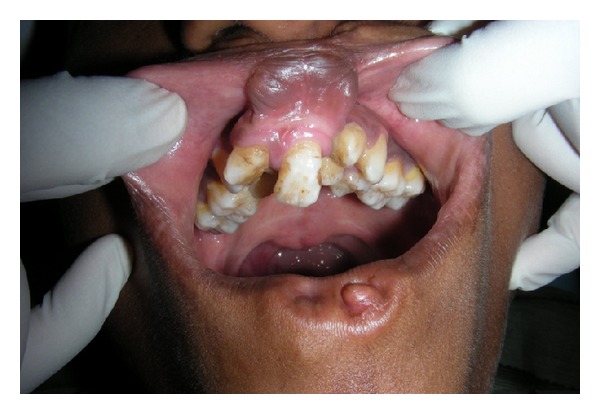
Crowding of teeth in upper arch along with fusion of the labial mucosa to the gingiva resulting in obliteration of labial vestibule.

**Figure 4:**
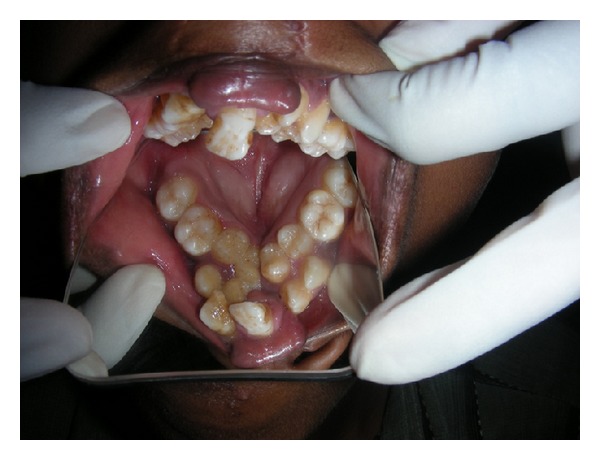
Collapsed high palatal arch.

**Figure 5:**
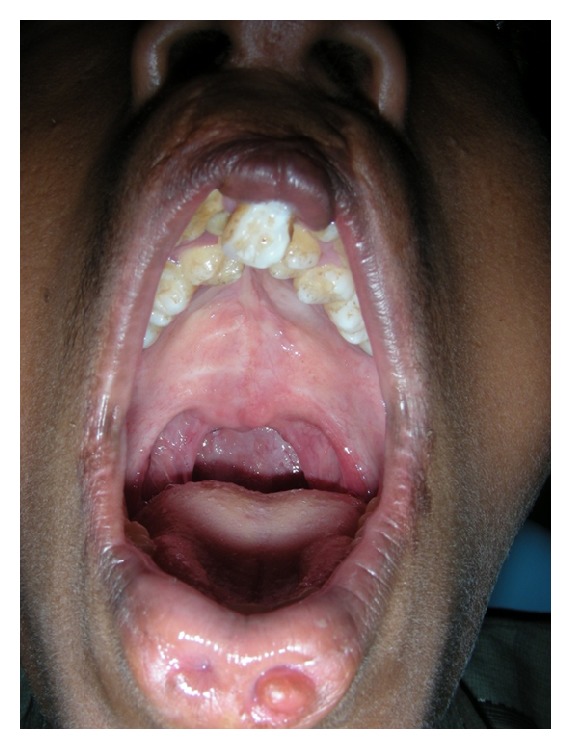
Short uvula.

**Figure 6:**
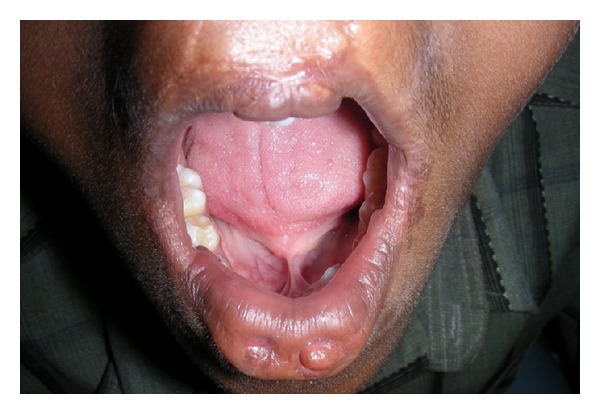
Ankyloglossia.

A fifteen-year-old boy reported to the Department of Oral Medicine and Radiology with a chief complaint of crowding of his upper and lower front teeth. Examination of the face revealed midface retrusion and surgically repaired bilateral upper cleft lip during his childhood with presence of small, bilateral, and paramedian lower lip pits (Figure 1). History revealed that he was the first born child to his parents. Antenatal history of the mother was negative for any significant illnesses and drug intake during pregnancy. Family history revealed positive consanguineous marriage of his parents and none of the family members had cleft lip/palate. Detailed examination of the lip revealed bilateral lip pits with one pit present at the base of nipple-like elevation (Figure 2). These lip pits were 3 mm in diameter and 5 mm deep. When the lower lip was compressed, mucous secretion was expressed from one of the lip pits. Intraoral examination revealed collapsed V-shaped palate with severe upper anterior teeth crowding (Figures 3 and 4). There was obliteration of the upper labial vestibule due to the adhesion of the lip to the gingiva by a thick fibrous band which blanched on retraction of the upper lip. Patient presented with a short uvula (Figure 5). However, mandibular teeth were all present and well aligned along with presence of short lingual frenum causing ankyloglossia (Figure 6). Posterior crossbite was noted which was attributed to the presence of narrow and collapsed arch. General medical examination was performed to rule out presence of systemic problems. Radiographic investigations like chest radiograph, lateral cephalograph, orthopantomograph, and maxillary occlusal view were taken. Genetic studies were not performed as the patient's parents did not give consent for the same.

The treatment planned for the patient was orthodontic treatment involving the correction of crowding of teeth and collapsed palatal arch along with cosmetic correction of the lip pits. Patient was also referred to an ENT surgeon for opinion regarding correction of tongue-tie and assessment of speech. Patient is waiting to undergo orthodontic treatment.

## 3. Discussion

One of the most common developmental defects seen in South India is cleft lip and palate. Among them, a few cases are associated with congenital lip pits and are termed as VWS [2]. Burdick and Bixler [4] presented the analysis of medical records of patients from the last 140 years (from 1845 to 1985). The descriptions of Van der Woude's teams involved 864 patients from 164 families living in France. Rizos and Spyropoulos [5] also reviewed cases of VWS from 1912 to 1999 in 91 cases reported in 56 published articles.

**Figure 7:**
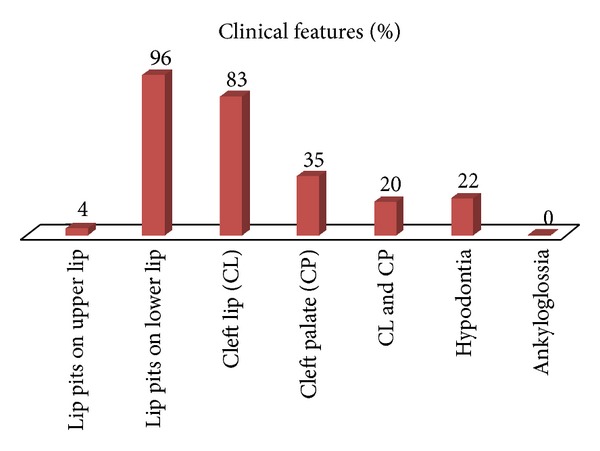
Showing the clinical features noted in all of the cases of VWS reviewed by the authors.

A short review was conducted by the authors. All of the cases of VWS reported from year 2002 to year 2013 were studied for the presence of cardinal features of the syndrome. The cardinal features of the reviewed cases have been tabulated in Table 1 and Figure 7.

### 3.1. Epidemiology

*Prevalence.* The incidence of VW syndrome is about 1 in 40,000 to 2,00,000 people [2, 3, 6–8].

*Gender Predilection.* Many authors believe that there is high prevalence of the syndrome in females. In the survey of 19 reports, the syndrome was predominantly noted in males. The syndrome is noted in male patient in the present paper.

### 3.2. Cardinal Features of VWS

Lower lip pits are the principal trait of VWS and are discussed here in relation to their location, morphology, symptomatology, aetiology, and histopathology.

*Location.* The typical presentation of lower lip pits is the bilateral paramedian sinuses of the lower lip placed symmetrically on either side of the midline. In the present review, most of the cases reported this presentation [2, 3, 9]. They can be unilaterally, medially, or bilaterally asymmetrical. Such a presentation is considered as an incomplete expression of the trait. The lip pits are usually circular or oval but can also be transverse slit-like or sulci. The transverse mucosal ridges, the conical elevations (nipple-like), and/or openings with no depth represent microforms of lower lip pits.

Nakano et al. [9] reported a case with midline fistula on the upper lip. According to the authors, the incidence of the fistula on lower lip is 0.001% and that of fistulas on upper lip is even lower.

*Morphology.* The lip pits form canals and are lined by labial mucosa which extends into the orbicularis oris muscle. They can present as two nipple-like protrusions with no sinus openings at their apices as presented in the case reported by the authors here. The canals always end as blind sacs surrounded by mucous glands. 

*Symptomatology.* Most of the times the lip pits are asymptomatic; the only symptom might be the continuous or intermittent drainage of watery or salivary secretion. In the present review, mucous type of secretion was noted in most reports [2, 3]. Rizos and Spyropoulos [5] in their review observed that there are rapid accumulation of mucous secretion on mastication and fear of apprehension, before or during mealtime. It was also reported that the secretion worsened during winter seasons in some patients.

*Morphogenesis.* The first report on lower lip pits by Demarquay attributed formation of lower lip pits to the impressions made on the lower lip by the upper central incisors. Most patients tend to adopt this hypothesis even now [5]. According to Kitamura, in a 32-day embryo, the lower lip consists of four growth centres, divided by one median and two lateral grooves. In the 38-day embryo, the lateral grooves disappear, except in the case of impeded mandibular process growth that results in the formation of a lip pit. If a cyst deriving from the epithelial wall communicated with the duct of labial glands, a congenital fistula of the lip is formed. The development of lip pits starts at day 36 of development, CL at day 40, and CP at day 50. The periods of liability of these three tissues probably vary in length and even in sequence, and perhaps they also overlap accounting for the strong association between the lip pits and cleft lip or palate [5, 10].

*Histopathology.* The pits reveal extensive depression in the central part, well surrounded by elevated borders. The stratified epithelium of the borders and the central area is thinned, while most of the basal cells are vacuolated with displacement of the nucleus resembling immature epithelial cells [5].

### 3.3. Associated Features of VWS

There are many associated features which may or may not be present in a case with the cardinal signs of the syndrome. The other associated features of VWS are hypodontia, hypoplasia, ankyloglossia, high arched palate, limb anomalies, congenital heart defects, and so forth. Dental abnormalities are uncommon in the syndrome. Among them, hypodontia is the one which is considered as a cardinal associated feature. It is observed in 10–20% of cases and occasionally represents the only expression of the gene. The lateral incisor and second molar are the most commonly affected teeth [1]. In the review presented by the authors, hypodontia was seen in 36% of the patients. The other associated features which were seen in the present review are hypoplasia [11], malocclusion, high arched palate, crossbite [12], bifid uvula [6, 12], and syngnathia [6]. Fibrous bands were noted bilaterally in the posterior region of the maxilla and mandible. These fibrous bands connected the alveolus of the maxilla and mandible in the region of the posterior maxillary tuberosity and posterior mandibular ramus area.

Ankyloglossia or tongue-tie is a congenital abnormality of the lingual frenulum. This entity is recognized but poorly defined condition and has been reported to cause feeding difficulties, dysarthria, dyspnoea, and social or mechanical problems. The exact pathophysiology of tongue-tie is unknown. The mucosa covering the anterior two-thirds of the mobile tongue is derived from the first pharyngeal arch and deviation of normal development is the most likely cause of abnormal frenulum length. One of the rare features associated with VWS is ankyloglossia which is reported by the authors in the present case. Ankyloglossia is a rare feature associated with VWS and is not commonly reported in the literature. Tongue-tie was reported by the authors in the present case [13].

### 3.4. Genetic Expressivity

The expression of the syndrome is variable; all of the signs can be present, either alone or in combination, or no abnormalities can be detected clinically. VWS is associated with deletion of chromosome 1q32–q41, but an extra chromosomal locus at 1p34 has been identified [2, 7]. Several studies have identified mutations in the gene encoding interferon regulatory factor 6 in VWS causing its marked variable expression [1, 14, 15]. Approximately 30–50 percent of all cases arise as* de novo* mutations [7].

### 3.5. Differential Diagnosis

VWS has a varied expressivity. The occurrence of different forms of VWS has to be kept in mind. There are many other syndromes which are considered as allelic variants of the syndrome and present with congenital lip pits like orofacial digital syndrome and popliteal syndrome. The following are considered in differential diagnosis of VWS [2, 16].

1. First is the popliteal pterygium syndrome (PPS) that includes popliteal web, CL and/or CP, lower lip pits in 60% cases, and anomalies of genitourinary system, such as cryptorchidism and bifid scrotum in males and hypoplastic labia majora and uterus in females. People with VWS have a risk of giving birth to offspring with PPS [11].
2. Second is Hirschsprung's disease (aganglionic megacolon combined with CP and lip pits) [17].
3. Third is orofacial digital syndrome type 1, with striking orodental, facial, digital, renal, and central nervous system abnormalities. Orodental signs include CP, bifid tongue, hypodontia, and median cleft of the upper lip and/or lip pits. This syndrome should be differentiated based on orodigital findings [18].
4. Finally, the following are, moreover, considered: ankyloblepharon filiforme adnatum—partial or complete full thickness fusion of the lid margins—cleft lip and palate, hydrocephalus, meningocele, imperforate anus, bilateral syndactyly, infantile glaucoma, and cardiac problems such as patent ductus arteriosus and ventricular septal defects [19].

### 3.6. Treatment

The primary indication for excision of congenital lip sinus is treatment of the associated cosmetic deformity, although it is acknowledged in the literature that many patients neither require nor request surgery [7]. There will be a small subset of patients where recurrent inflammation justifies excision of the lip sinus tracts [5]. Excision of the sinus tract should be complete, because if some of the mucous glands attached to the fistula are left behind, this could allow a mucoid cyst to form. Loosening of the lip muscle has also been reported as a drawback of the operation [20]. There has been a single case report of a carcinoma developing in the area of the lower lip and this may have been secondary to a chronic inflammatory process [21].

## 4. Conclusion

A rare case of VWS is reported by the authors. The rare feature noted in the present case is presence of ankyloglossia which is one of the additional features seen in VWS but has not been reported in patients. The multidisciplinary approach for the treatment of the present case was undertaken, which involved the role of plastic surgeon, an ENT surgeon, and various disciplines of dentistry. The presence of salivary fistulae diagnostic for Van der Woude syndrome is not the important medical problem. Much more significant is the probability of developing cleft defects by the offspring of the patients, which reaches 67%; hence, this finding is of great significance for genetic counselling [22].

## Figures and Tables

**Table 1:** Summary of the presence of lip pits, cleft lip and/or palate, hypodontia, and ankyloglossia in Van der Woude syndrome patients presented in the respective literature.

S. No	Reference	Gender	Lip Pits	Cleft lip (CL)	Cleft palate (CP)	CL & CP	Hypodontia	Ankyloglossia
1	Arangannal et al., (2002) [3]	M	*✓*	*✓*			*✓*	
2	Surasak et al., (2003) [6]	F	*✓*	*✓*	*✓*	*✓*		
3	Moore and McCord (2004) [23]	M	*✓*	*✓*	*✓*	*✓*		
4	Souissi et al., (2004) [7]	M	*✓*	*✓*				
5	Rizos and Spyropoulos (2004) [5]	F	*✓*	*✓*				
6	Stanier and Moore (2004) [24]	F	*✓*	*✓*				
7	King et al., (2004) [25]	M	*✓*	*✓*				
8	Karande and Patil (2005) [16]	M	*✓*	*✓*			*✓*	
9	Tokat et al., (2005) [14]	M	*✓*	*✓*				
10	Newman et al., (2005) [11]	F	*✓*	*✓*			*✓*	
11	Ziai et al., (2005) [26]	M	*✓*					
12	Kirzioglu and Ertürk (2006) [27]	M	*✓*	*✓*				
13	King et al., (2004) [25]	F	*✓*		*✓*		*✓*	
14	Klinische Padiatrie (2008) [28]	F	*✓*	*✓*	*✓*	*✓*		
15	Etöz O. A. and Etöz A. (2009) [29]	M	*✓*					
16	Nakano et al., (2010) [9]	M	*✓* (UL)	*✓*				
17	Lam et al., (2010) [30]	M	*✓*	*✓*	*✓*	*✓*	*✓*	
18	Baghestani et al., (2010) [31]	M	*✓*					
19	Moghe et al., (2010) [8]	F	*✓*	*✓*	*✓*	*✓*		
20	Jobling et al., (2011) [15]	M	*✓*	*✓*	*✓*	*✓*		
21	Manoharan et al., (2013) [10]	M	*✓*	*✓*				
22	Shweta et al., (2012) [2]	M	*✓*	*✓*				

CL: cleft lip, CP: cleft palate, and UL: upper lip.
